# The current state and trends of immunotherapy research in lung cancer: a review and bibliometric analysis

**DOI:** 10.3389/fonc.2024.1428307

**Published:** 2024-11-11

**Authors:** Yun-Hua Zheng, Li Chen, Xiang Liu, Rong-Hui Li, Hai-Bo Lei, Guang-Hui Chen

**Affiliations:** ^1^ Department of Quality Evaluation and Medical Record Management, The Affiliated Hospital of Southwest Jiaotong University and The Third People’s Hospital of Chengdu, Chengdu, Sichuan, China; ^2^ Department of Clinical Pharmacy, Xiangtan Central Hospital, Xiangtan, Hunan, China

**Keywords:** immunotherapy, lung cancer, bibliometric analysis, visual analysis, CiteSpace

## Abstract

In recent years, the integration of immunotherapy in the treatment of lung cancer has marked a significant evolution in the field. This is evidenced by the surge in the volume of scientific publications, reflecting rapid advances over time. This paper presents a bibliometric analysis of lung cancer and immunotherapy research from January 2012 to December 2022, drawing on the Web of Science literature database and using the citexs data analysis platform to examine the shifts in topic hotspots over the decade. A total of 8,722 publications were retrieved, with annual publication numbers soaring from 79 in 2012 to 2,112 in 2021. The most prolific country in terms of publication volume was China (n = 3,363, 38.56%), with The University of Texas MD Anderson Cancer Center making the most significant institutional contribution (n = 156, 1.79%). Notably, the most productive authors in this domain were Benjamin Besse and Marina Chiara Garassino, who have collectively published 35 articles to date. Predominant research themes include PD1/PDL1, clinical trials, pembrolizumab, nivolumab, and immune checkpoint inhibitors. Moreover, this paper visualizes the analysis of journals, keywords, key genes and targets, and associated diseases, aiming to provide a systematic review and a forward-looking perspective on research in lung cancer and immunotherapy. By exploring current research dynamics and hotspots and identifying areas for improvement, this study seeks to provide valuable insights for future investigations in this burgeoning field.

## 1 Introduction

Over the past few decades, lung cancer has consistently ranked among the most common cancer diagnoses worldwide and is the leading cause of cancer mortality (1). This complex disease is primarily categorized into two types: non-small cell lung cancer (NSCLC), which constitutes the majority (approximately 85%), and the less prevalent small cell lung cancer (SCLC), accounting for about 15% (2). Treatment modalities encompass surgical resection, radiation therapy, and chemotherapy. Notably, the advent of immune checkpoint inhibitors (ICIs) and targeted therapies in recent years has significantly enhanced the survival prospects of patients (3). The PD-1/PD-L1 immune pathway serves as a critical target in therapy. ICIs activate T cells by blocking this pathway, thereby amplifying the immune response against tumors (4). Monotherapy with anti-PD-(L)1 agents or their combination with other treatments have become a standard treatment regimen for most lung cancer patients, especially those at advanced stages (5).

Entering the 2010s, the field of lung cancer immunotherapy experienced rapid expansion, driving a significant yearly increase in research publications. Researchers have increasingly focused on inducing T cell-mediated antitumor responses. One such T cell-mediated therapeutic approach is chimeric antigen receptor T cell immunotherapy (CAR-T), which targets any cell surface molecule without HLA restriction and has been successfully applied in treating hematologic malignancies (6, 7). It is crucial, albeit challenging, to track the dynamics of these studies and to capture key developments promptly. Thus, an in-depth and extensive quantitative analysis of research findings is necessary to systematically review critical scientific advances, highlight emerging research areas, and recommend future research directions.

Bibliometric analysis, a powerful tool for assessing the overall state of a discipline through the quantitative analysis of a large body of structured information on publications, is employed to understand the status, trends, and hotspots of a given field of study (8, 9). With its growing popularity in the medical domain, bibliometric analysis is increasingly applied to analyze research dynamics in lung cancer immunotherapy (10, 11). Although there have been bibliometric studies of lung cancer immunotherapy, they either cover incomplete periods or are limited in scope and fail to encompass the full spectrum of immunotherapy (12). However, this analysis focuses on original articles published on clinical immunotherapy for lung cancer from January 2012 to December 2022. By conducting a comprehensive analysis of publication trends, research countries/regions, institutions, authors, journals, research domains, citation networks, keyword frequency hotspots, temporal shifts in keyword popularity, associated gene clustering, protein-protein interaction (PPI) network construction, and associated disease clustering, it aims to unveil the latest research hotspots. Based on this study, researchers can swiftly identify progress, clarify research directions, select collaboration partners, determine target journals for publication, and summarize future research hotspots in this field.

## 2 Materials and method

### 2.1 Database and retrieval strategy

The authors opted for the Web of Science database to conduct a search for publications related to immunotherapy in lung cancer. Recognized for its applicability in bibliometric analysis, this database encompasses over ten thousand influential journals and provides comprehensive citation data (13, 14). Furthermore, the accuracy of the document type labels within the Web of Science has been established as superior to other databases (15). A literature search was conducted on April 1, 2023, with restrictions on original research articles published between 2012 and 2022. The search strategy was meticulously defined: (1) Keywords were searched exclusively in titles, as irrelevant papers might include the search keywords in their abstracts; (2) The keywords used were “lung cancer” and “immunotherapy”; (3) Synonyms for the keywords were included as comprehensively as possible, with synonyms for “immunotherapy” encompassing specific names of drugs or treatments; (4) Non-original research papers, including reviews and meta-analyses, were excluded. Multiple tests and adjustments were performed to refine the search strategy for precision. Duplicates were eliminated by comparing the Digital Object Identifier (DOI) and the PubMed Unique Identifier (PMID) of the publications. A total of 9,915 documents were retrieved, with only articles published in English being included (N = 8,872). The extracted data encompassed titles, abstracts, keywords, authors, countries/regions, institutions, journals, publication dates, and total citation counts.

### 2.2 Statistic analysis

Bibliometric indicators, including the number of publications, publication year, type, funding information, countries, institutions, authors, keywords, associated diseases, and related genes, were exported as BibTeX and analyzed using Microsoft Excel (Redmond, WA, USA) spreadsheets and VOSviewer software for capture and calculation. GraphPad Prism 9 software (Dotmatics, San Diego, CA, USA) was employed for the creation of histograms and bubble charts. Bibliometric analysis and data visualization were conducted using the R software (v4.1.2) with Bibliometrix (16). VOSviewer facilitates the construction of co-occurrence matrices, culminating in the visualization of networks, including network, density, and overlay visualizations (17). CiteSpace software (v6.1.R1) was utilized to detect keywords and references with the strongest citation bursts, create visualizations of co-cited references and keywords, and generate dual-map overlays of journals. To delineate and visualize research trends in immunotherapy for lung cancer from 2012 to 2022, the authors categorized articles by searching for specific therapies and treatment lines in the titles and abstracts. Microsoft Visual Basic for Applications was used to execute macros for data arrangement and batch retrieval. The networks of countries, authors, institutions, and keywords were visualized and explored based on their co-occurrence. In the figure, items were represented by their annotations and circles or frames, with the size of the item labels determined by the frequency of the item occurrence-the higher the occurrence rate, the larger the item label. The proximity between two items in the visualization approximates their relevance in terms of co-citation links. In overlay visualizations, items were distinguished by color, determined by the publication year, with the color bar displayed at the bottom right of the visualization. The darker shades of purple represented more recent times. Nodes or frames of varying sizes denoted the frequency of keyword occurrences, facilitating the identification of hotspots in leading-edge terminology.

## 3 Result

### 3.1 Analysis of annual publication trend

**Figure 1:**
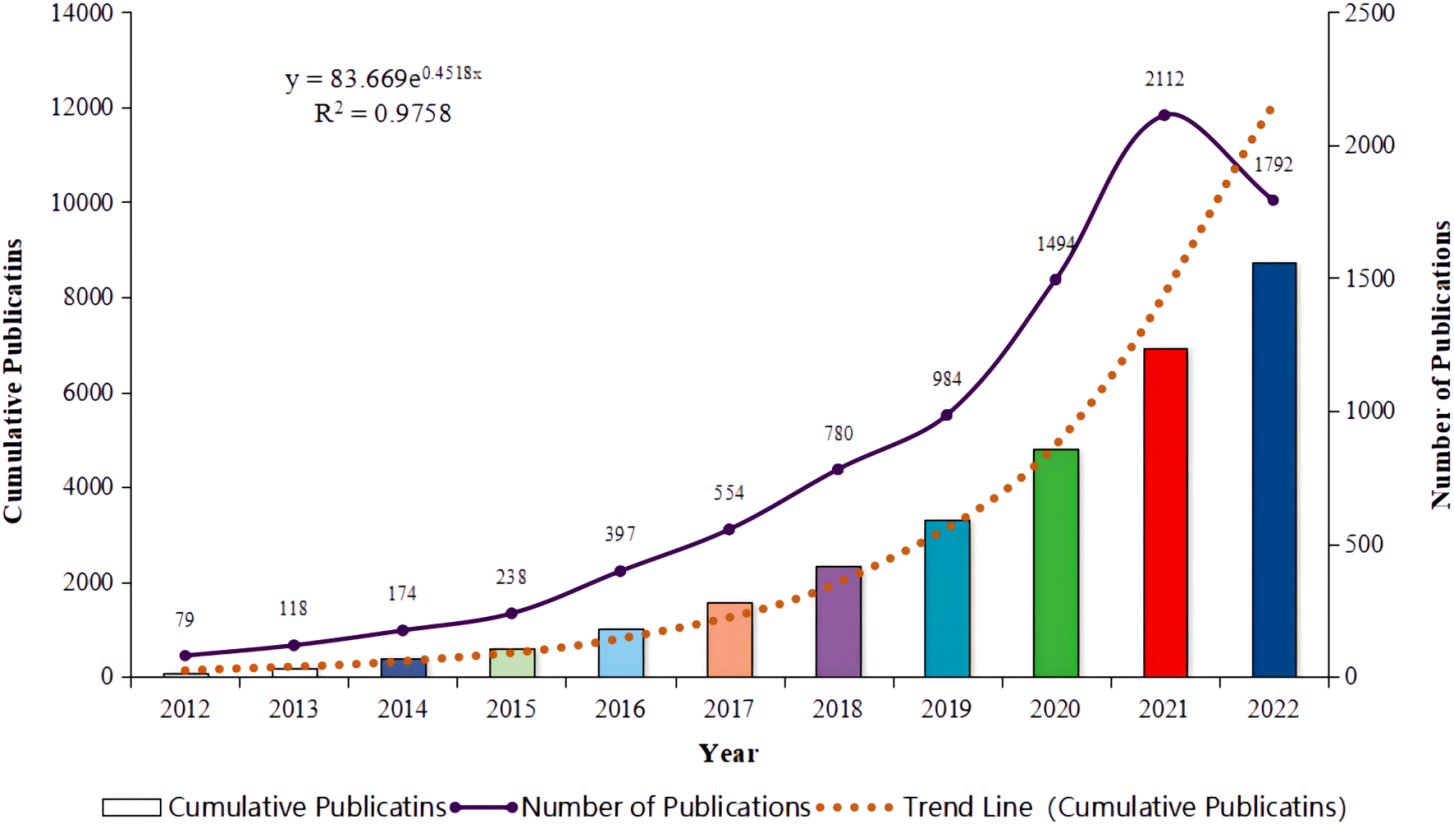
Annual publication trends for literature related to lung cancer and immunotherapy from January 2012 to December 2022.

Between 2012 and 2022, a total of 8,722 publications were retrieved, with an average annual publication volume of 793 articles. As illustrated in **Figure 1**, the annual publication volume experienced a rapid increase from 79 publications in 2012 to 2,112 in 2021, nearly 26 times the volume of publications in 2012. The year 2016 witnessed the fastest growth rate at 66.81%, indicating rapid development in the field and a phase of swift ascent. However, a downward trend was observed in 2022, with the number of publications dropping to 1,792 articles, which merits further investigation.

### 3.2 Analysis of countries and institutions

**Figure 2:**
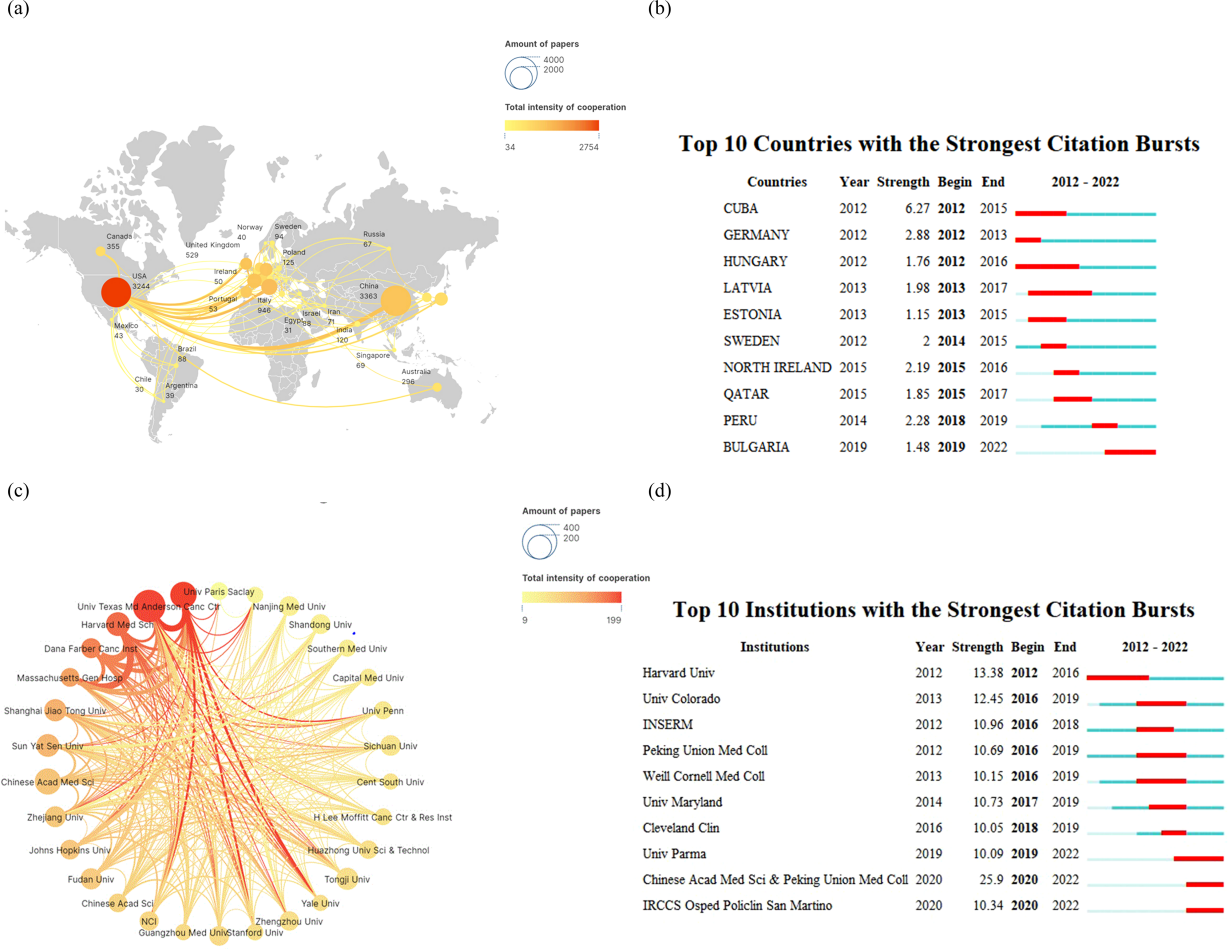
Analysis of research regions **(A)** for lung cancer and immunotherapy from January 2012 to December 2022, countries with the strongest citation bursts **(B)**, relationship analysis of research institutions **(C)**, and institutions with the strongest citation bursts **(D)**.

From 2012 to 2022, over 20 different countries were significantly involved in the publication of a vast array of articles on immunotherapy for lung cancer, as depicted in **Figure 2A**. The top five most active countries accounted for over 90% of the publications. China led the way, contributing more than a third of all records (n = 3,363, 38.56%), followed by the United States (n = 3,244, 37.19%), Italy (n = 946, 10.85%), the United Kingdom (n = 529, 6.07%), and Germany (n = 355, 4.07%). The top 30 of all universities or institutions contributing to research on immunotherapy in lung cancer are shown in **Figure 2C**. The University of Texas MD Anderson Cancer Center and Memorial Sloan Kettering Cancer Center topped the list with 156 and 124 publications, respectively, followed by Sichuan University at No. 3 with 112 publications. Funding agencies in China and the United States remain the most active in this field. Furthermore, as illustrated in **Figures 2B, D**, we summarized the ten countries and ten institutions with the strongest citation bursts in the lung cancer immunotherapy domain. Bulgaria emerged as the latest hotspot country. Similarly, recent hotspot research institutions include the University of Parma, the Chinese Academy of Medical Sciences with Peking Union Medical College, and IRCCS Osped Policlin San Martino.

### 3.3 Analysis of study authors, journals and literature co-citation

**Figure 3:**
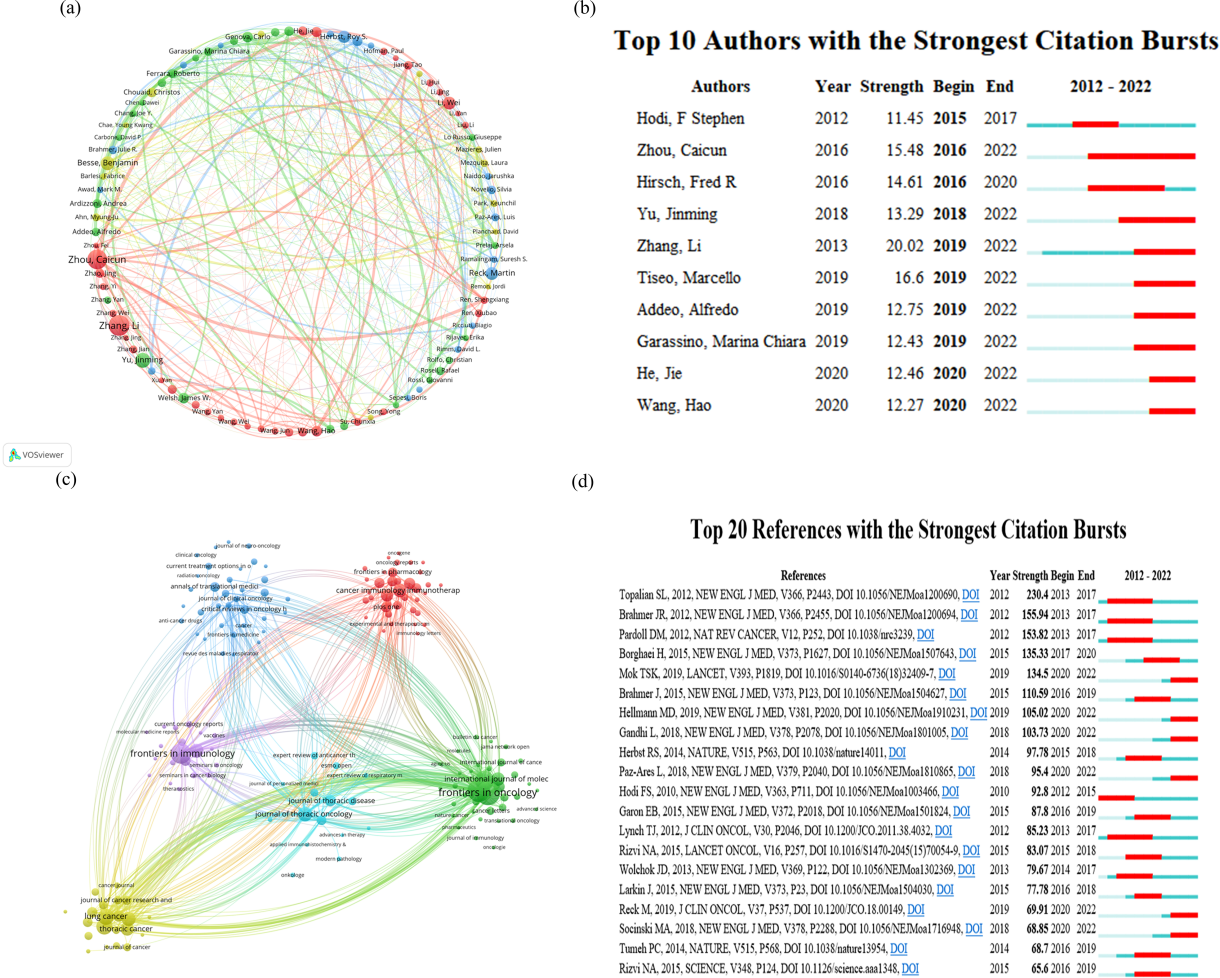
Analysis of research author relationships **(A)** for lung cancer and immunotherapy from January 2012 to December 2022, citation burst of authors **(B)**, journal publication clustering **(C)**, and citation burst of literature **(D)**.

**Figure 3A** depicts the global authorship of lung cancer and immunotherapy research from January 2012 to December 2022. The most prolific authors in this field were Benjamin Besse and Marina Chiara Garassino, who have collectively published 35 articles to date. Diego Signorelli and Giuseppe Lo Russo were close behind with 30 publications each, while Jie He was third with 28. The journals that have published the most on lung cancer immunotherapy are illustrated in **Figure 3C**. These journals clustered into six groups, led by Frontiers in Oncology, Frontiers in Immunology, Cancer Immunology Immunotherapy, Lung Cancer, Journal of Thoracic Oncology, and Annals of Translational Medicine, which have been the most prolific in the field over the past decade. Moreover, as shown in **Figure 3B**, we summarized the ten authors with the strongest citation bursts in lung cancer immunotherapy at different time points. The most recent prominent researchers identified were Jie He and Hao Wang. The New England Journal of Medicine (N Engl J Med) emerged as the most influential journal in this domain, with many publications appearing in this outlet, as evidenced in the top 20 most cited documents (**Figure 3D**).

### 3.4 Analysis of keywords, hotspot word frequency, and associated disease clustering

**Figure 4:**
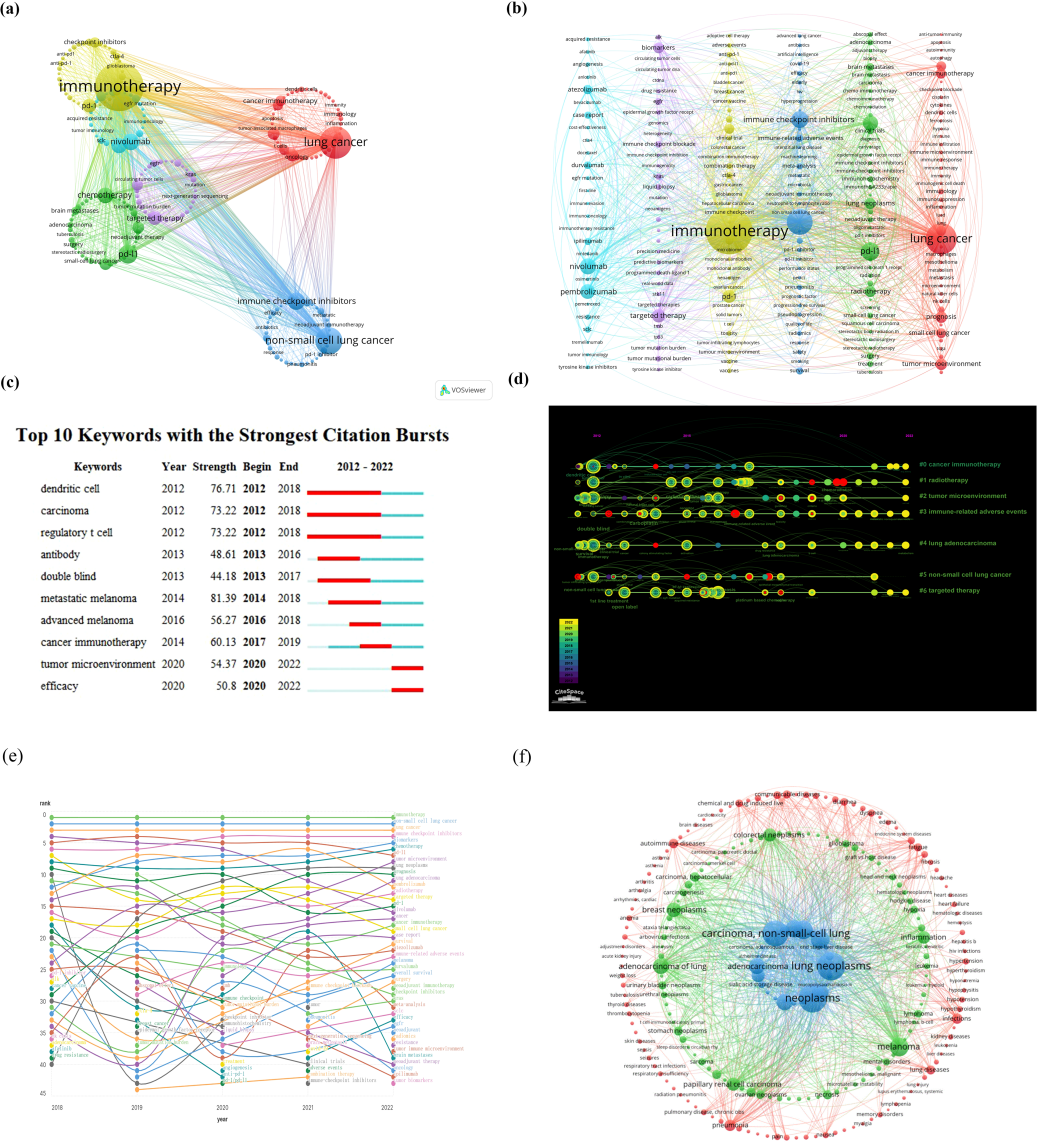
**(A, B)** Clustering analysis of keyword frequency hotspots, **(C, D)** citation burst of hotspot keywords and the timeline view for co-cited references, **(E)** analysis of keyword popularity rankings and ranking changes over different time periods, **(F)** clustering analysis of associated diseases.

Keywords in a paper serve as a concise and comprehensive encapsulation of research purpose, topic, and methodology. An analysis based on these keywords can reflect the thematic evolution trends and research hotspots within a certain field over a specific period. Employing “lung cancer” and “immunotherapy” as search keywords for the period from January 2012 to December 2022, **Figures 4A, B** illustrate the clustering analysis of keyword hotspot frequencies. Keywords related to lung cancer and immunotherapy were clustered into six groups. The most frequently occurring keywords included “immunotherapy,” “lung cancer,” “chemotherapy,” “PD-L1,” “targeted therapy,” “non-small cell lung cancer,” “nivolumab,” “pembrolizumab,” “PD-1,” and “immune checkpoint inhibitors.” This analysis identified the top 10 most cited keywords in the literature on immunotherapy for lung cancer (**Figure 4C**), visually demonstrating the periods with higher publication volumes for different hotspot keywords. **Figures 4D, E** depict the ranking and rank variation of keyword frequencies associated with lung cancer and immunotherapy across various time periods. The top 10 keywords from 2018 to 2022 remained nearly unchanged, in order: immunotherapy, non-small cell lung cancer, lung cancer, immune checkpoint inhibitors, biomarkers, chemotherapy, PD-L1, tumor microenvironment, and prognosis. A total of 8,722 papers were retrieved using “lung cancer” and “immunotherapy” as search keywords from January 2012 to December 2022. The BioBERT biomedical language representation model was employed to mine and statistically cluster the disease entities mentioned in the abstracts of these articles. As shown in **Figure 4F**, the highest number of publications was related to neoplasms (7,620 articles) and lung neoplasms followed with 6,970 articles. Carcinoma of the non-small cell lung was third with 5,677 articles. Moreover, diseases such as adenocarcinoma of the lung, stomach neoplasms, papillary renal cell carcinoma, melanoma, inflammation, colorectal neoplasms, and breast neoplasms also featured prominently in terms of publication volume.

### 3.5 Analysis of associated gene clustering and construction of protein-protein interaction networks

**Figure 5:**
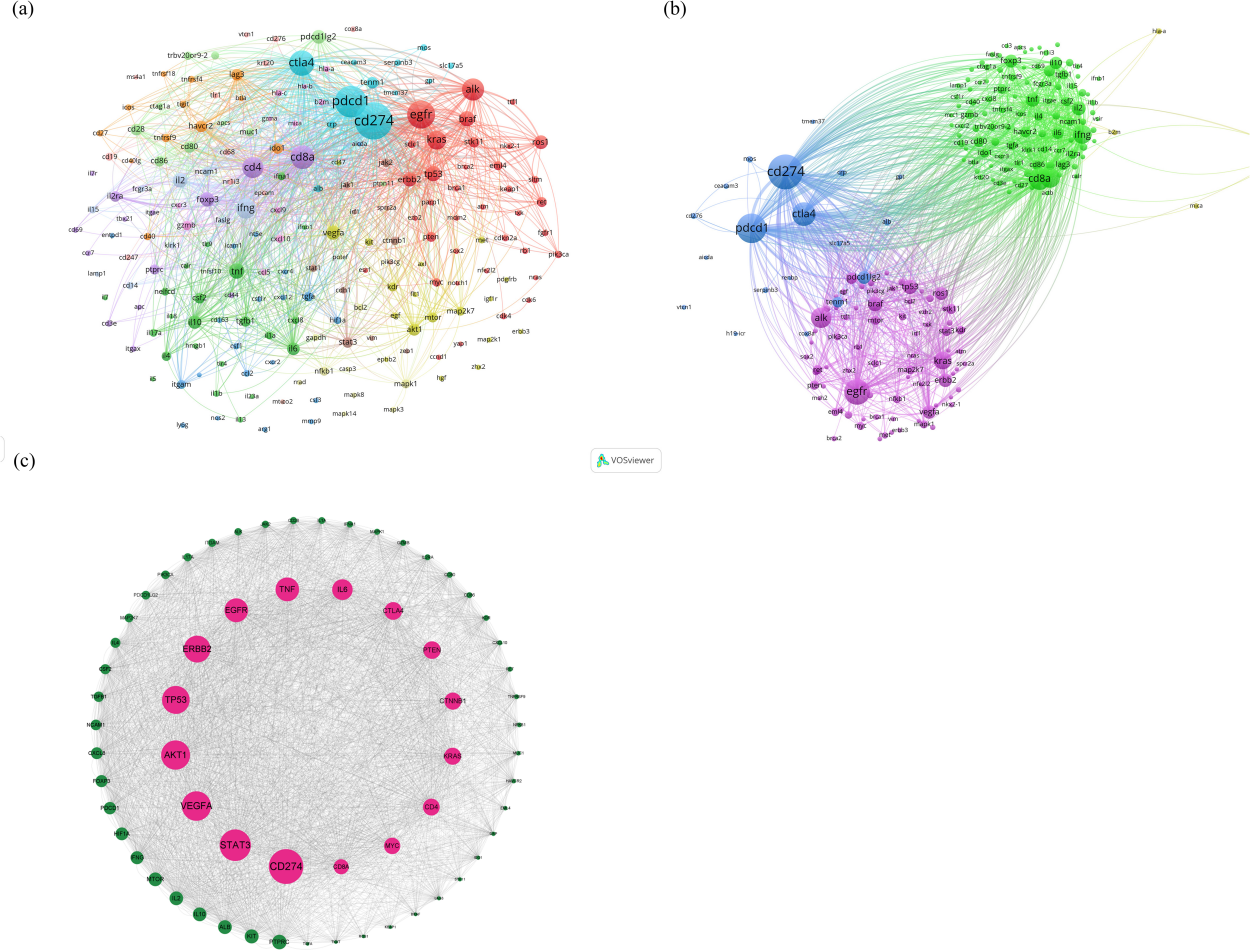
**(A, B)** Clustering analysis of genes associated with lung cancer and immunotherapy from January 2012 to December 2022, **(C)** analysis results of the construction of the PPI network.

The BioBERT biomedical language representation model was applied to mine and statistically analyze the gene entities mentioned in the abstracts of these articles. **Figures 5A, B** present the gene clustering analysis associated with lung cancer and immunotherapy for this period, highlighting CD274 as the gene most frequently mentioned across the literature (4,608 articles). PDCD1 and EGFR followed with 2,798 and 2,607 mentions, respectively. In addition, the top 10 associated genes included CTLA4, ALK, CD8A, CD4, KRAS, IFNG, and FOXP3. The construction of the protein-protein interaction (PPI) network, illustrated in **Figure 5C**, identifies the top ten proteins as CD274, STAT3, VEGFA, AKT1, TP53, ERBB2, EGFR, TNF, IL6, and CTLA4. These proteins are likely core to the study of immunotherapy in lung cancer, suggesting their pivotal roles in the research landscape.

## 4 Discussion

### 4.1 Global trends in immunotherapy for lung cancer

Given the substantial global burden of the disease, lung cancer has consistently been a highly esteemed research domain (18). A quantitative and comprehensive review of research trends, status, and hotspots in immunotherapy for lung cancer is presented, based on the bibliometric analysis of thousands of articles. “Lung Cancer” emerges as the most productive journal in the realm of lung cancer immunotherapy, whereas “The New England Journal of Medicine” stands as the most influential publication in this field. Researchers from China have contributed a significant portion of the studies, yet papers with corresponding authors from the United States tend to be more impactful. Most top-level papers are authored by international collaborations spanning multiple countries/regions. Despite the publication of some studies from developing countries in recent years, there remains a noticeable lack of research from Africa or the Middle East. The University of Texas MD Anderson Cancer Center was identified as the most prolific institution. While several universities in China have made contributions to numerous articles, their TPR is low. Conversely, several institutions from developed countries, despite a lower overall publication count, have contributed many top-tier articles.

### 4.2 Hotspots and emerging trends in immunotherapy for lung cancer

An examination of keyword frequencies, hot-spot terms, and disease clustering suggests that prior to 2015, the bulk of lung cancer immunotherapy publications focused on vaccine strategies. The number of publications on vaccination or adoptive cell therapy (ACT) demonstrated minimal annual fluctuations. Since 2013, there has been a notable increase in the number of publications discussing ipilimumab. From 2015 onwards, anti-PD-1 antibodies have taken center stage in research efforts, closely followed by anti-PD-L1 antibodies. The advent of immune checkpoint inhibitors has significantly altered the landscape of research in cancer immunotherapy. Recent studies have been assessing the efficacy of first-line immunotherapy or combination therapies as opposed to second-line immunotherapy or monotherapy (19). Adjuvant and neoadjuvant treatments have recently become areas of intense research interest (20). Combining radiotherapy with immune checkpoint inhibitors (ICIs) has surfaced as a new research hotspot. Among the ICIs, nivolumab, durvalumab, and pembrolizumab, when used in combination with radiotherapy, have been the subject of the most substantial evaluation compared to other ICIs (21–24).

#### 4.2.1 Pembrolizumab

Pembrolizumab, an immune checkpoint inhibitor targeting the PD-1 receptor, has been recognized for its capacity to potentiate antitumor immunity. As per established therapeutic protocols, it is mandatory for all patients diagnosed with metastatic non-small cell lung cancer (NSCLC) to undergo immunohistochemistry (IHC) testing to determine PD-L1 expression and the presence of other oncogenic driver mutations, including EGFR, ALK, ROS-1, and BRAF, prior to the initiation of treatment regimen (25). For those patients with metastatic NSCLC who are in a physical condition graded between 0 to 2 and exhibit no contraindications to immunotherapy, pembrolizumab is advised as the initial monotherapy of choice, provided that the PD-L1 expression is equal to or greater than 50%, and there is an absence of driver mutations. When PD-L1 expression ranges from 1 to 49% and all driver mutations are tested negative, pembrolizumab is recommended as a front-line therapy in combination with chemotherapeutic agents. Specifically, pembrolizumab in combination with carboplatin or cisplatin and pemetrexed is preferred in cases of non-squamous carcinoma or adenocarcinoma. For patients with squamous cell histology, a regimen of pembrolizumab in combination with carboplatin and a taxane (either paclitaxel or albumin-bound paclitaxel) is preferred (26).

The KEYNOTE-024 trial was designed to evaluate the efficacy of pembrolizumab monotherapy against platinum-based chemotherapy in NSCLC patients whose tumors expressed PD-L1 at levels of 50% or greater and who did not harbor the driver mutations. The monotherapy with pembrolizumab resulted in an improved median survival (30.0 months vs. 14.2 months) (27). The KEYNOTE-189 trial, a phase III study targeting patients with metastatic non-squamous NSCLC, compared the outcomes of pembrolizumab in combination with carboplatin or cisplatin and pemetrexed against chemotherapy alone, revealing a superior one-year overall survival (OS) rate for the combination therapy cohort (69.2% vs. 49.4%) (28). Similarly, the KEYNOTE-407 trial, another phase III study focusing on patients with metastatic squamous cell NSCLC, assessed the effectiveness of pembrolizumab in conjunction with carboplatin and paclitaxel or nab-paclitaxel, and found an enhancement in median survival for patients receiving combination therapy (15.9 months vs. 11.3 months) (29).

#### 4.2.2 Durvalumab

In the Phase III randomized PACIFIC trial, the effectiveness of durvalumab as consolidation immunotherapy was evaluated in the context of adjuvant treatment compared to a placebo in patients with unresectable stage III NSCLC who had previously undergone concurrent chemoradiation therapy. The trial’s findings demonstrated a significant enhancement in both overall survival (OS) and progression-free survival (17.2 vs. 5.6 months) (30).

#### 4.2.3 Nivolumab

Nivolumab and ipilimumab are immunotherapeutic agents functioning as immune checkpoint inhibitors, with complementary mechanisms of action on T cells. Nivolumab targets the PD-1 receptor, while ipilimumab acts as a CTLA-4 blocking antibody in humans. In the Phase III randomized trial CheckMate 227, the efficacy of first-line therapy with nivolumab/ipilimumab was assessed in comparison to nivolumab monotherapy and chemotherapy in patients with metastatic NSCLC, with a high tumor mutation burden (TMB ≥ 10 mutations per megabase) and no driver mutations present. The study reported a median progression-free survival (PFS) advantage for the combination therapy group (7.2 months vs. 5.5 months) (31).

As subsequent therapy in the Phase III randomized trial CheckMate 057, the efficacy of nivolumab was compared to docetaxel as a follow-up treatment during or after first-line chemotherapy for patients with metastatic non-squamous NSCLC. The trial indicated a superior median survival (MS) result for the nivolumab group (12.2 months vs. 9.4 months) (32). In addition, nivolumab was compared with docetaxel as a follow-up treatment in patients with metastatic squamous cell NSCLC who had experienced disease progression following chemotherapy in the Phase III randomized trial CheckMate 017. This report demonstrated better MS outcomes for the nivolumab group (9.2 months vs. 6 months) (33).

Beyond immune checkpoint inhibitors (ICIs), immunotherapeutic strategies against lung cancer also encompass vaccination. The clinical utility of various therapeutic vaccines has been demonstrated, such as TG 4010, BLP25, and NEO-PV-01 (34–38). Despite their demonstrated tolerability and low toxicity, cancer vaccination faces multiple challenges including limited infiltration in tumors, changing immune responses over time, and the emergence of resistance. Multivalent vaccines targeting epitopes optimized for immunogenicity may address some of these challenges. A deeper understanding of immune evasion mechanisms, the design of efficacious formulations, and the combination with other immunotherapies targeting the tumor microenvironment (TME) and tumor-derived factors can accelerate the development of next-generation cancer vaccines. Current vaccine research distinguishes between antigen-specific vaccines (peptide/protein vaccines, DNA vaccines, and vector-based vaccines) and whole-cell vaccines (allogeneic and autologous dendritic cell vaccines) (39–42).

Adoptive cell therapy (ACT) has demonstrated antitumor activity and can serve as a salvage therapy for certain lung cancer patients. In recent years, TIL-based ACT has been evaluated for lung cancer. A Phase I trial reported that TIL therapy was safe and exhibited profound and durable responses in some patients (43). Moreover, ACT employing chimeric antigen receptor (CAR) modified cells has also demonstrated antitumor immunity in some studies (44, 45). While ACT based on novel technology presents a promising salvage therapy for some lung cancer patients, response rates have been suboptimal and clinical evidence is lacking. Further research is needed to identify patients who are good candidates for ACT and to provide more substantial evidence.

### 4.3 Targeted study in immunotherapy for lung cancer

The analytical integration of associated gene clustering and protein-protein interaction (PPI) network construction has identified top-ranking genes and pivotal therapeutic targets. Scientists have effectively exploited the specificity of antibodies to target tumor antigens on the surface of cancer cells, leading to the development of a plethora of targeted antibodies that diminish the activity of tumor cells. These therapeutic antibodies are generally classified into three categories: (1) monoclonal antibodies; (2) antibody-drug conjugates (ADCs); (3) bispecific antibodies. In the past two decades, several monoclonal antibodies have been FDA-approved for the treatment of non-small cell lung cancer (NSCLC), including cetuximab, bevacizumab, nivolumab, and pembrolizumab (**Table 1**). Cetuximab is an anti-EGFR monoclonal antibody that selectively binds to the extracellular domain of EGFR, thereby interfering with its receptor tyrosine kinase (RTK)-mediated downstream proliferative signaling. This antibody has been shown to elicit favorable responses in a variety of combination therapeutic regimens. Additional anti-EGFR monoclonal antibodies under investigation for NSCLC include necitumumab, nimotuzumab, and ficlatuzumab (46–49). Bevacizumab, an anti-VEGF monoclonal antibody, has demonstrated anti-angiogenic properties that inhibit tumor growth and was the inaugural drug of its class to receive FDA approval. Ramucirumab, another anti-VEGF agent, has exhibited significant therapeutic potential in NSCLC when used in combination therapy protocols (50).

**Table 1:** Targeted antibodies for lung cancer therapy.

Targeted Antibodies	Lung cancer type	Related Molecule	Target/Bioactivity	Reference
Monoclonal Antibodies (MABs)	NSCLC	Cetuximab	Anti-EGFR	(46)
		Necitumumab	Anti-EGFR	(47)
		Nimotuzumab	Anti-EGFR	(48)
		Ficlatuzumab	Anti-EGFR	(51)
		Bevacizumab	Anti-VEGF	(52)
		Ramucirumab	Anti-VEGF/VEGFR2	(50)
		Nivolumab	Anti-PD-1	(53)
		Pembrolizumab	Anti-PD-1	(27)
		Ipilimumab	Anti-CTLA-4	(54)
		Tremelimumab	Anti-CTLA-4	(55)
		Denosumab	Anti-RANKL	(56)
		Figitumumab	Anti-IGF-1R	(57)
	SCLC	Tarextumab	Anti-Notch2/Notch3	(58)
		Tucotuzumab	Anti-EpCAM	(59)
		Bec2	Anti-GD3	(60)
Antibody–Drug Conjugate (ADC)	SCLC	Rovalpituzumab tesirine	Anti-DLL3	(61)
		Sacituzumab govitecan	Anti-Trop-2	(62)
		Lorvotuzumab mertansine	Anti-CD56	(63)
	NSCLC	Ado-Trastuzumab emtansine	Anti-HER2	(64)
		Telisotuzumab vedotin	Anti-cMET	(65)
		Enapotamab vedotin	Anti-AXL	(66)
Bispecific antibodies	NSCLC	Amivantamab	Anti-EGFR, Anti-MET	(67)

## 5 Challenges and prospects

Immunotherapeutic strategies have shown considerable promise in the lung cancer treatment arena, yet they are still in the embryonic stage of development. A salient challenge is the absence of optimized, uniformly standardized *in vitro* and *in vivo* laboratory models, as well as preclinical and clinical frameworks, which are imperative for the assessment of efficacy, underlying mechanisms, pharmacodynamics, and toxicity profiles of immune-targeting agents (68). Customarily, murine models with intact immune systems, such as genetically engineered mouse models (GEMMs), chemically-induced models, and syngeneic tumor transplant models have been employed, which have already partially addressed certain questions (69, 70). However, they do not provide the thorough representation necessary to decipher the fundamental tenets of immunobiology or to evaluate novel immunotherapeutic interventions. Humanized mice, or mice engrafted with human immune systems (HIS), are increasingly leveraged in preclinical investigations of immunotherapy despite their exorbitant cost (71). The development of organoid or tumoroid models with 3D co-cultures of immune cells and organ-on-a-chip models based on microfluidics for using patient-derived tumor cells has significant potential but is still in early stages of development. Consequently, there is an urgent need for the development of dependable models that can illuminate the principal factors of cancer immunity, immune response mechanisms, therapeutic modalities, mechanisms of both primary and secondary immune evasion, synthetic immunity, and toxicological implications inherent in the translational application of cancer immunotherapies.

Further inquiry is paramount to a more nuanced understanding of the various immunological facets of lung cancer, including mechanisms of immune evasion, immunosuppression, immune editing, and the tumor’s intrinsic adaptive responses to the stress induced by immunotherapy, with the aim of reviving and guiding patient immunity against neoplastic diseases with precision. Although combination immunotherapies have demonstrated efficacy, a consensus on the selection of therapeutic regimens remains elusive. Prospective treatment modalities for non-small cell lung cancer (NSCLC) may involve an amalgamation of chemotherapy, neoantigen vaccine administration, and the deployment of multiple immune checkpoint inhibitors (ICIs) to engage reactivated tumor signaling pathways. The concomitant administration of nivolumab and ipilimumab has emerged as a potent immunotherapeutic combination for patients with advanced NSCLC. The potential of various novel immunomodulators in the context of combination therapy remains largely uncharted. Ongoing clinical trials are dedicated to ascertaining the safety, tolerability, and efficacy of both monotherapies and combination immunotherapies. The intricate interplay between epigenetic and genetic alterations is a driving force in the pathogenesis of pulmonary neoplasms. Alterations in the epigenetic landscape can precipitate dysregulation of oncogenes and tumor suppressor genes, culminating in increased cellular proliferation, hastened cell cycle progression, resistance to apoptosis, and modulation of immune responses (72). Epigenetic therapies currently center on the use of DNA methyltransferase inhibitors (DNMTis), histone deacetylase inhibitors (HDACis), Janus kinase 2 inhibitors, and RNA-based treatments, in conjunction with immunotherapy to enhance the curative potential of lung cancer treatments and to prolong patient survival (73). By targeting both the intrinsic tumor and the tumor ecosystem-associated adaptive stress pathways (including metabolic, oxidative, endoplasmic reticulum, DNA damage, and replication stress responses), therapeutic strategies can be devised that are translatable to clinical applications. Immunotherapy can be synergistically combined with epigenetic inhibitors, such as DNMTis, HDACis, and EZH2 inhibitors, to target epigenetically mediated tumorigenesis and immune inhibition.

The armamentarium of adoptive cell therapy (ACT) for the treatment of lung cancer is expanding. Significant strides have been made in clinical arenas with therapies such as chimeric antigen receptor T cells (CAR T), T cell receptor (TCR) engineered cells, and tumor-infiltrating lymphocytes (TIL), though challenges persist. Chemokines play a pivotal role in the recruitment and infiltration of T cells into pulmonary neoplasms, with heightened infiltration levels of CD4+ and CD8+ T lymphocytes being prognostically favorable (74). Jin et al. have recently deployed CAR T cells expressing CCR6 in a xenograft mouse model targeting lung cancer, demonstrating promising T cell infiltration and tumoricidal activity (75). Adachi and colleagues engineered CAR-T cells to express interleukin-7 and CCL19 (crucial for the sustenance of T-cell zones within lymphoid organs), yielding encouraging outcomes in mouse models (76). In addition, the augmentation of multi-omics platforms at the single-cell level and the accessibility of publicly available datasets can greatly enrich our understanding of the immune landscape and thus inform treatment choices.

Personalized immunotherapeutic approaches in lung cancer warrant investigation. Tumor heterogeneity may present a significant bottleneck in the design of personalized immunotherapy. Dynamical properties of the immune microenvironment and spatiotemporal analysis of different types of lung cancer can shed light on the immune repertoire, antigen presentation modalities, and immune editing processes. Advances in multiplexed immunofluorescence (MIF), imaging mass cytometry (IMC), chip-cytometry, multiplexed ion beam imaging (MIBI), DNA barcode-based mIHC/IF, and *in-situ* multiplex analysis techniques are instrumental in deciphering the tumor immune repertoire (77). Whole-exome sequencing (WES), RNA-seq, single-cell sequencing, and TCR sequencing can expose the tumor mutanome and cell-specific TCRs with neoantigen specificity, augmenting cancer vaccination strategies (78). Challenges arise from factors such as TCR diversity, TCR clonality, neoantigen clonality, neoantigen subtypes, and differential artist metrics. Each patient’s cancer cells harbor a unique neoantigen-MHC complex mixture, referred to as the neoantigenome. Multi-component AI-based computational algorithms can examine the binding complementarity of mutant peptides to patient HLA alleles to assess the potential to elicit anti-tumor T cell responses. AI methods such as TSNAD, pVAC-Seq, INTEGRATE-neo, NetMHCpan, MARIA, EDGE, and DeepHLApan employ multi-layer architectures to discern patterns and predict the efficacy and immunogenicity of MHC-I/II binding and neoantigen presentation (79, 80). While computational binding predictions can yield informative insights, LC-MS/MS analysis of MHC molecule immunoprecipitation and peptide identification will generate an accurate and robust database, such as the Immune Epitope database (IEDB) (81). Personalized neoantigen identification facilitates the development of next-generation immunotherapies. For preclinical and clinical applications, the predicted binding affinity of neoantigens to their corresponding MHCs, with affinities greater than 500 nM, is considered immunogenic epitopes and subsequently selected for the development of tailored cancer vaccines (82). The discovery of lung cancer-specific composite biomarkers is imperative for the classification of tumor immunogenicity, patient stratification, pharmacodynamic prediction, and ultimately the determination of regulatory endpoints to enhance the efficacy of immunotherapy for lung cancer. Recent advancements in AI-based algorithms and models will soon be able to predict responses to immunotherapy and combination therapies at an individual level, further enabling the effective clinical utilization of immunotherapies (83).

Although immunotherapy holds promise as a potent method for treating cancer due to its ability to evoke immune memory, and clinical data suggests better tolerance with minimal side effects, immune-related adverse events (irAEs) are observed in some patients (84). Side effects can range from flu-like symptoms, rashes, pain, edema, palpitations, diarrhea, and an over-activated immune state to organ system damage. Cytokine release syndrome and onset of diabetes have also been observed in patients undergoing CAR-T and immunotherapy. Interstitial and alveolar infiltration following pneumonia are the most common pulmonary irAEs (85). While immunosuppressive corticosteroids are the treatment of choice for irAEs, there is evidence that they may impair the efficacy of immunotherapies. The study of immunomodulatory nanomaterials and nutraceuticals may pave new pathways to address irAEs and autoimmune toxicity. Further research is necessary to better understand the mechanisms of IRAEs to effectively manage adverse reactions in lung cancer immunotherapy.

## 6 Conclusion

The therapeutic landscape for patients with advanced lung cancer has been fundamentally altered by the advent of immunotherapy and continues to evolve. Based on an analysis of 8,722 publications, this study provides researchers with an exhaustive overview of research trajectories, historical progression, and current hotspots in lung cancer immunotherapy, while also providing inspiration for future investigations. Several therapeutic options are available for patients with advanced lung cancer, ranging from monotherapy to quadruple therapy, which combines immunotherapy with chemotherapy and anti-angiogenic agents. The United States Food and Drug Administration (FDA) has approved the solitary use of immunotherapeutic agents or their combination with other immunotherapies and chemotherapy for the treatment of advanced lung cancer, as delineated in this article. Significant studies have influenced the field by demonstrating the efficacy of various immunotherapies in improving survival rates for advanced lung cancer patients. Key advancements include the identification of critical pathways and the development of inhibitors targeting these checkpoints, which have become cornerstone treatments in the field. The emergence of resistance to immune checkpoint inhibitors (ICIs), whether intrinsic or acquired, poses a significant challenge in oncology. Cell therapy represents a potential and pragmatic addition to the armamentarium of lung cancer immunotherapies. Despite the lack of tumor-specific antigens, a hostile tumor microenvironment (TME), and toxicity concerns, cell therapy remains an attractive, albeit undoubtedly challenging, prospect. Clinical studies have evaluated innovative therapeutic approaches, including combinations of PD-1/PD-L1 inhibitors with various ICIs and DNA repair-targeting drugs, and sequencing strategies. Overall, the therapeutic advantages of ICIs and ACTs suggest a favorable trajectory for the future of effective lung cancer treatment. This study highlights the importance of continuous research and collaboration in overcoming the challenges of resistance and optimizing therapeutic strategies to improve patient outcomes.

## Data Availability

The original contributions presented in the study are included in the article/Supplementary Material. Further inquiries can be directed to the corresponding authors.
